# Towards a Better Understanding of Cohesin Mutations in AML

**DOI:** 10.3389/fonc.2019.00867

**Published:** 2019-09-09

**Authors:** Sergi Cuartero, Andrew J. Innes, Matthias Merkenschlager

**Affiliations:** ^1^Faculty of Medicine, MRC London Institute of Medical Sciences, Institute of Clinical Sciences, Imperial College London, London, United Kingdom; ^2^Centre for Genomic Regulation (CRG), The Barcelona Institute of Science and Technology, Barcelona, Spain; ^3^Josep Carreras Leukaemia Research Institute (IJC), Barcelona, Spain; ^4^Faculty of Medicine, Centre for Haematology, Imperial College London, London, United Kingdom

**Keywords:** cohesin, leukemia, interferon, inflammation, hematopoiesis, AML

## Abstract

Classical driver mutations in acute myeloid leukemia (AML) typically affect regulators of cell proliferation, differentiation, and survival. The selective advantage of increased proliferation, improved survival, and reduced differentiation on leukemia progression is immediately obvious. Recent large-scale sequencing efforts have uncovered numerous novel AML-associated mutations. Interestingly, a substantial fraction of the most frequently mutated genes encode general regulators of transcription and chromatin state. Understanding the selective advantage conferred by these mutations remains a major challenge. A striking example are mutations in genes of the cohesin complex, a major regulator of three-dimensional genome organization. Several landmark studies have shown that cohesin mutations perturb the balance between self-renewal and differentiation of hematopoietic stem and progenitor cells (HSPC). Emerging data now begin to uncover the molecular mechanisms that underpin this phenotype. Among these mechanisms is a role for cohesin in the control of inflammatory responses in HSPCs and myeloid cells. Inflammatory signals limit HSPC self-renewal and drive HSPC differentiation. Consistent with this, cohesin mutations promote resistance to inflammatory signals, and may provide a selective advantage for AML progression. In this review, we discuss recent progress in understanding cohesin mutations in AML, and speculate whether vulnerabilities associated with these mutations could be exploited therapeutically.

## Introduction

Hematopoietic homeostasis requires tight regulation to ensure production of sufficient numbers of blood cells at all stages of differentiation. This is achieved by a complex network of signaling pathways and gene regulatory mechanisms that control cell proliferation, differentiation, and survival of hematopoietic stem and progenitor cells (HSPC) and their progeny. Skewing of this balance in favor of excessive differentiation results in stem cell depletion, exhaustion and eventually, inability to replenish mature blood cells. In contrast, uncontrolled self-renewal, increased survival, and failure to differentiate are hallmarks of leukemia.

The homeostatic balance between self-renewal and differentiation of HSPC is sensitive to a broad range of perturbations. Mutations that disrupt it not only provide classifiers of clinical disease, but also offer insights into the molecular control of self-renewal, differentiation, and cell proliferation. Many AML-associated mutations are clearly linked to one of these categories, such as constitutive activation of RAS proteins or FLT3, that drive uncontrolled proliferation (1–3), mutations that prevent cell cycle arrest and apoptosis such as TP53 (4), and mutations that hinder differentiation such as in the transcription factors RUNX1 or C/EBPα (5, 6). The clonal advantage conferred by such mutations is immediately obvious.

**Figure 1:**
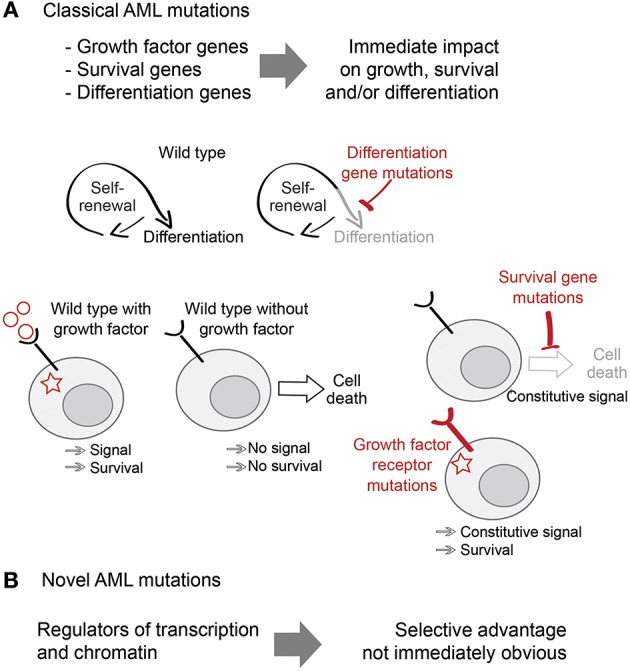
Classical and non-classical AML mutations. **(A)** Classical AML mutations deregulate proliferation, survival and differentiation pathways and provide an obvious selective advantage to AML. **(B)** Novel AML mutations include mutations in transcription and chromatin regulators and their selective advantage is less obvious.

Recent large-scale sequencing studies have shown that the mutational landscape of AML is highly enriched for mutations in general transcriptional regulators and chromatin modifiers, which are found in ~70% of patients (7). Examples of this group include mutations in proteins involved in chromatin modifications (ASXL1, EZH2), DNA methylation (DNMT3A, TET2), or transcriptional splicing (SRSF2, U2AF1) (7–9). Although we understand the biological functions of many of these molecules and pathways in exquisite detail, their selective advantage for AML cells remains largely unknown (10). Understanding the link between these novel AML mutations and the molecular mechanisms of self-renewal, differentiation and cell survival is critical for understanding the pathophysiology of AML, and for the identification of new therapeutic approaches to cancer (Figure 1).

A striking example are mutations in the subunits of the cohesin protein complex (SMC1, SMC3, RAD21, and STAG1/2). Cohesin forms a ring-shaped structure that can encircle DNA and hold sister chromatids together. This function of cohesin is essential for DNA replication (11–13), DNA repair (14–17), and chromosome segregation in mitosis (18–20). Despite this essential role in cell cycle progression, heterozygous or hypomorphic cohesin mutations are compatible with cell proliferation (21). This explains how leukemic cells can tolerate cohesin mutations, but fails to explain why cohesin mutations occur with high frequencies in AML.

In addition to essential functions in the cell cycle, cohesin has a major role in three-dimensional genome organization (22). Cohesin cooperates with the DNA-binding protein CTCF in the formation of topologically associated domains (TADs), which facilitate preferential interactions between genes and enhancers within the same CTCF-demarcated domain (23–26). Impaired formation of these structures randomizes the three-dimensional topology across single cells (27), thus exposing genes and enhancers to illegitimate interactions (28).

Here we review recent progress that links impaired cohesin function to the regulation of inflammatory gene expression, self-renewal, and differentiation of hematopoietic progenitors (29–34), revealing potential explanations for why cohesin is recurrently mutated in AML. We speculate about the role of inflammatory gene expression in AML and its potential therapeutic implications.

## Cohesin Mutations in AML

Mutations in members of the cohesin complex are found in 6–13% of AML cases (7, 35–37). In addition to AML, cohesin mutations are also found in other myeloid malignancies, including Down syndrome acute megakaryoblastic leukemia (DS-AMKL), myelodysplastic syndrome (MDS), myeloproliferative neoplasms (MPN), chronic myelomonocytic leukemia (CMML), and chronic myelogenous leukemia (CML) (38, 39). Cohesin mutations are frequent in myeloid but not in lymphoid malignancies, suggesting that the pro-leukemic effect of reduced cohesin function may be connected to myeloid-specific traits.

The majority of RAD21 and STAG2 mutations cause non-sense, frame-shift, or splice-site changes, presumably leading to protein truncation or exon skipping. On the other hand, mutations in SMC1A and SMC3 are missense, causing amino acid substitutions in different protein domains (38). The effect of each of these mutations on the formation of the cohesin complex is still largely unexplored. Some of the mutant transcripts can give rise to dominant-negative proteins in cord blood progenitors (32) while others result in the degradation of the mutant transcript (38).

Most cohesin mutations are heterozygous, consistent with the idea that complete loss of the complex is incompatible with cell cycle progression. This has been confirmed by studies showing that partial cohesin loss in AML cells is not linked to increased aneuploidy (29, 36, 38, 40). However, since the *Stag2* and *Smc1a* genes are on the X chromosome, male cases with mutations in these genes are not heterozygous. In the case of STAG2 mutant cells it has been shown that STAG1 becomes essential (41), suggesting that loss of STAG2 can be at least partially compensated by STAG1.

Cohesin mutations in patients appear to be mutually exclusive, indicating that a mutation in just one member of the complex is sufficient to reduce cohesin activity to the point where it provides a clonal advantage. Cohesin mutations often co-occur with mutations in other genes, such as NPM1, TET2, ASXL1, and EZH2 (36, 38). Nonetheless, it is thought that the majority of cohesin mutations are early events in leukemogenesis (38, 42). The prognostic significance of cohesin mutations in myeloid malignancies is not yet fully clear. In MDS, STAG2 mutations are associated with significantly reduced survival (37). However, no significant association between cohesin mutations and survival in AML was found in an early study (36) while a more recent study reports a significant association with increased overall survival and disease-free survival (43).

## The Role of Cohesin Early Hematopoiesis

The frequency of cohesin mutations in AML prompted several groups to investigate the contribution of cohesin to early hematopoiesis and myeloid differentiation (Table 1). A mouse model of conditional *Smc3* heterozygosity (31) presented an altered composition of the hematopoietic stem cell (HSC) compartment. Short-term HSCs and multipotent progenitor populations (MPP) were increased, while in long-term HSCs were decreased (31). In competitive repopulation assays, cohesin-deficient cells outcompeted wild-type cells. An important aspect of this study was the demonstration that *Smc3* heterozygosity on its own is not sufficient to trigger leukemic transformation. However, the combination of *Smc3* heterozygosity and an internal duplication in the FLT3 receptor (one of the most common mutations in AML) induced acute myeloid leukemia in mice. This indicates that SMC3 mutations must cooperate with other mutations to cause leukemia in this model. However, a mouse model of conditional *Stag2* deletion presented features of myeloid dysplasia (44). Also, these mice had increased frequencies of both long-term and short-term HSC, indicating that mutations in different cohesin subunits do not always cause the same phenotypes.

**Table 1:** Main phenotypes and transcriptional changes in models of cohesin depletion in hematopoiesis.

**Model**	**Main phenotypes of cohesin depletion**	**Transcriptional changes**	**References**
Mouse model of conditional Smc3 heterozygous deletion Mx1-Cre; Smc3^lox/WT^	Increase in ST-HSC and MPP and decrease in LT-HSC Increased bone marrow chimerism in competitive transplantation assays Absence of leukemic transformation unless combined with Flt3 mutations Increased self-renewal in methylcellulose assays	Deregulation of lineage-specific transcription factors Global reduction in transcription	(31)
Mouse model of conditional Stag2 deletion Mx1-Cre; Stag2^lox/lox^	Features of myelodysplasia Increase in ST-HSC, LT-HSC, MPP, and GMP Increased bone marrow chimerism in competitive transplantation assays Blockade in B-cell differentiation	Downregulation of lymphoid, myeloid and erythroid lineage commitment genes Downregulation of genes involved in HSC quiescence	(44)
Mouse model of doxycycline-inducible shRNA expression for Rad21, Smc1a, and Stag2 TRE-shRNA; ROSA26^(M2rtTA/+)^	Decrease in ST and LT-HSC and increase in GMP Myeloid disorder features (splenomegaly and myeloid hyperplasia) Increased self-renewal in methylcellulose assays	Upregulation of myeloid differentiation genes (Fcgr3, Cebpa) Downregulation of lymphoid development genes (Blnk, Lax1, Cd86)	(30)
Human CD34+ cord blood cells transduced with mutant cohesin genes (RAD21^E212^^*^, RAD21^Q592^^*^, SMC1A ^R711G^)	Impaired sensitivity to cytokine-induced differentiation Increase in CD34+ progenitors after engraftment in NSG mice Increased self-renewal in methylcellulose assays	Upregulation of HSC genes (HOX genes, MEIS1) Downregulation of myeloid differentiation genes (MPO, CSF1R)	(32)
Human CD34+ cord blood cells transduced with shRNA^STAG2^	Delayed differentiation and expansion of immature cells over time after engraftment in NSG mice Increased self-renewal in methylcellulose assays	Upregulated genes enriched in HSC-specific genes	(33)
Mouse HSPCs transduced with shRNA^Rad21^	Increased self-renewal in methylcellulose assays	Increased expression of the self-renewal genes HoxA7 and HoxA9	(29)
Rad21^−/−^ mouse macrophages and Rad21^+/−^ HSPCs	Defective inflammatory response Decrease in LPS-induced differentiation	Impaired inducible gene expression Downregulation of tonic interferon expression	(34)
Mouse HSPCs transduced with shRNA^Rad21^ and mouse models of conditional Rad21 deletion Mx-Cre; Rad21^lox/+^	Decrease in LPS-induced differentiation Positive selection of Rad21-deficient HSCs during aging	Inhibited NFkB transcriptional response	(45)

* *indicates nonsense mutations*.

Mouse models of shRNA-mediated knock-down of different cohesin subunits developed similar, but not identical alterations in stem cell compartments (30). In the bone marrow there was a marked increase in granulocyte-macrophage progenitors (GMP), accompanied by a decrease in long-term and short-term HSCs. These models of cohesin deficiency did not develop acute myeloid leukemia. However, the mice displayed several features resembling a myeloid disorder, including splenomegaly and myeloid hyperplasia. In addition, cohesin-mutated mouse cells acquire increased self-renewal capacity in *in vitro* methylcellulose colony formation assays.

Importantly, similar results were obtained with human cells (32, 33). Cohesin-deficient cord blood progenitors or AML cell lines displayed reduced sensitivity to the differentiation-inducing effects of cytokines. The same effect was observed by over-expressing cohesin genes carrying mutations identified in AML, indicating that these can act as dominant-negative mutants. These cells were also characterized by a higher frequency of CD34^+^ progenitors and increased self-renewal capacity in methylcellulose (32). In line with these findings, cohesin-deficient human blood progenitors have increased *in vivo* reconstitution capacity after transplantation into immunodeficient mice (33).

## Transcriptional Consequences of Cohesin Mutations in HSPCs

Given that AML-associated cohesin mutations do not affect genome integrity (38), the observed resistance to differentiation has been ascribed to the gene regulatory role of cohesin. In all models tested, transcriptional changes were mild (31, 32). This is expected, as even complete removal of cohesin only changes the expression of 10% of genes (23).

Previous reports suggested a role for cohesin in facilitating chromatin remodeling in human and mouse cells (46, 47). Consistent with this, cohesin-deficient HSPCs present genome-wide alterations in chromatin accessibility (30–33, 45). These changes broadly correlate with altered gene expression. Therefore, it has been proposed that defective chromatin accessibility impacts on normal dynamics of transcription factor binding, which leads to transcriptional deregulation and abnormal differentiation.

An extreme case of chromatin alterations was observed in human cord blood cells, where dominant negative cohesin mutations reduced chromatin accessibility genome-wide (32). Interestingly, a minority of sites displayed increased accessibility, specifically binding sites for the transcription factors GATA2 and RUNX1. This has been proposed to result in an upregulation of HSPC transcriptional programs and obstruct differentiation. A role for cohesin in regulating RUNX1 expression has also been described in model organisms (48).

How is cohesin linked to chromatin accessibility? Cohesin binding sites are highly accessible (49). In yeast, cohesin cooperates with the chromatin structure remodeling complex (RSC) to actively evict nucleosomes and generate nucleosome-free DNA (50–52), which is required for cohesin loading (53). In mouse embryonic stem cells, depletion of a member of the PBAF complex (a vertebrate ortholog of RSC) results in sister-chromatid cohesion defects (54). In humans, cohesin is found in a complex with the ATP-dependent chromatin remodeling enzyme SNF2H (55). SNF2H, but not cohesin, is required for the establishment of arrays of phased nucleosomes around CTCF binding sites (56).

Reduced cohesin dosage can also alter the frequency of chromatin interactions near transcription factor genes. One example is the transcriptional regulation of the lymphoid transcription factor *Ebf1*, that has four STAG2 binding sites in hematopoietic progenitors. In *Stag2*^−/−^ mice, cis-interactions at this locus are lost, leading to abrogation of Ebf1 expression and failure to differentiate into lymphoid progenitors (44).

Another mechanism that has been proposed to explain the increased self-renewal capacity of cohesin-deficient HSPCs is the derepression of the self-renewal transcription factor HOXA9 (29, 57). HOXA9 is normally silenced by the Polycomb complex, which represses Hox loci in HSPCs by H3K27 trimethylation. In cohesin-deficient mouse HSPCs, this repressive chromatin mark is lost and *Hoxa9* is upregulated, leading to increased self-renewal. This finding suggests that cohesin cooperates with Polycomb to silence *Hox* genes in HSPCs. Consistently, in mouse embryonic stem cells, cohesin complexes containing STAG2 (but not STAG1) contribute to the maintenance of chromatin interactions within Polycomb domains (58).

## Cohesin in the Control of the Inflammatory Response

As discussed in the previous section, all studies that have compared wild-type and cohesin-deficient HSPCs found clear changes in chromatin accessibility and gene expression. These changes may indicate a direct effect of cohesin, or, alternatively, they may reflect the less mature state of cohesin-deficient progenitor populations. In order to rule out this possibility and determine what genes are directly controlled by cohesin in myeloid cells, a recent study used terminally differentiated macrophages to allow a like-for-like comparison of transcriptional and chromatin state between wild-type and cohesin-deficient cells (34). This strategy uncovered a key role for cohesin in the regulation of inflammatory gene expression. Consistent with a body of knowledge demonstrating that cohesin is required for interactions within topological domains (23–26), interactions between upstream key transcriptional regulators of the inflammatory response and their surrounding enhancers were decreased after acute cohesin depletion. As the organization of the inflammatory response is hierarchical, reduced levels of upstream regulators impact on the network, and deregulation spreads to the majority of inducible genes. Importantly, re-analysis of HSPC gene expression data showed that cohesin also controls inflammatory gene expression in progenitor cells (34).

Inflammatory signals not only mediate cross-talk between immune cells to coordinate the immune response, but also regulate the balance between HSPC self-renewal and differentiation. This function, known as emergency hematopoiesis, is normally activated during infection in order to regenerate mature myeloid cell populations (59). Several inflammatory cytokines and ligands, including interferons, are involved in the activation of emergency hematopoiesis. Type I interferon induces HSC exit from quiescence, entry into the cell cycle and differentiation. Importantly, chronic exposure to type I interferon is detrimental to HSCs (60, 61). Type II interferon—or IFNγ–also regulates HSC activity both in homeostasis and during infection (62). IFNγ acts on a subset of HSCs to induce myeloid differentiation by activating transcription factors like C/EBPβ (63). The interleukin IL-1 brings about myeloid differentiation through activation of a NF-κB-PU.1 axis (64). Activation of Toll-like receptor (TLR) signaling in HSPCs, which activates both the NF-κB and the interferon pathways, also promotes myeloid differentiation (65–69). As in the case of chronic interferon exposure, sustained TLR activation becomes detrimental and impairs the repopulating capacity of HSPCs.

**Figure 2:**
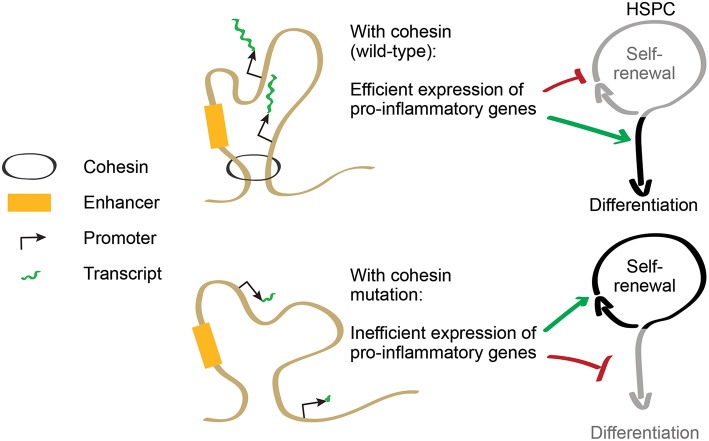
Cohesin regulates the balance between self-renewal and differentiation. Cohesin controls expression of pro-inflammatory genes that promote HSPC differentiation. In cohesin-mutant AML, inflammatory gene expression is downregulated, increasing resistance to differentiation and favoring HSPC self-renewal.

As cohesin is required to induce expression of inflammatory response genes, cohesin-deficient HSPCs are less prone to differentiate in inflammatory conditions [(34, 45); Figure 2]. This acquired resistance to differentiation allows increased proliferation of immature progenitors, providing a possible explanation for some of the phenotypes displayed by cohesin-deficient mice. Therefore, cohesin mutations in AML illustrate a mechanistic connection between the control of transcriptional regulation and the responsiveness to differentiation-inducing stimuli in myeloid cells. The selective advantage of mutations in other transcriptional regulators may potentially be explained by similar mechanisms involving the control of inflammatory signaling.

## Inflammatory Gene Expression in AML

Consistent with the finding that cohesin regulates inflammatory gene expression in hematopoietic progenitors and myeloid cells, AML patient cells with cohesin mutations show a striking reduction of inflammatory and interferon pathways. This is the case when comparing AML with and without cohesin mutations across all samples in The Cancer Genome Atlas (TCGA), as well as within a specific histological subtype (34). These data suggest that the same mechanism that favors self-renewal in cohesin-deficient HSPCs through impaired sensitivity to inflammatory signals may operate in cohesin-deficient AMLs. The implication is that in settings with increased inflammatory signaling, cohesin mutations could confer resistance to inflammatory signals and increased self-renewal and clonal expansion.

Constitutively increased inflammatory signals are a hallmark of aging. Basal levels of pro-inflammatory cytokines such as IL6 or TNFα increase with age in healthy individuals (70). This leads to alterations in hematopoietic differentiation, which are reminiscent of emergency hematopoiesis: myelopoiesis-biased differentiation and reduced HSC self-renewal (71, 72). Consistent with its role in conferring resistance to inflammatory signals, competitive assays show that cohesin mutant HSCs become dominant over wild-type HSCs in aged mice (45). As clonal hematopoiesis is a feature of aging (73), it has been suggested that cohesin mutations could be positively selected during aging, eventually promoting a pre-leukemic state (45). This is in line with a report showing that cohesin mutations are early events, considered to be pre-leukemic (42). However, cohesin subunits are not among the top frequently mutated genes in cases where clones of hematopoietic cells carrying somatic mutations are found in the absence of any hematologic dysplasia, known as clonal hematopoiesis of indeterminate potential (CHIP) (73). Therefore, the emergence of cohesin mutations associated to aging may immediately lead to pre-leukemic dysplasias rather than CHIP. Further investigation is required to understand the role of cohesin mutations during aging.

A number of previous studies have reported altered expression of cytokines and other inflammatory mediators in myeloid disorders (74–76). For example, *FLT3-ITD*^+^ AML show increased expression of microRNA miR-155, which is known for its anti-inflammatory effects, its ability to inhibit interferon signaling, and to increase HSPC self-renewal in mouse models (77). Sensitivity of human AML cells to IFNγ is inversely related to RIP1/3 signaling. High levels of RIP1/3 signaling stabilize SOCS1, and SOCS1 antagonizes IFNγ signaling, effectively protecting AML cells from the differentiation-inducing effects of IFNγ (78). On the other hand, reduced RIPK3 expression is thought to reduce differentiation and TNFR-driven death of AML cells (79). Interleukin-1 (IL-1) inhibits growth of normal hematopoietic progenitors, but promotes expansion of AML cells by increasing p38 MAPK phosphorylation. This effect can be reversed by blocking IL-1 with p38 MAPK inhibitors (80).

As an illustration of the complexity of the pathways involved in the regulation of inflammation and differentiation, TNF activates NFκB via JNK in AML cells (81) and can dampen interferon signaling via SOCS1 (78). NFκB is constitutively activated in CD34^+^CD38^−^ AML cells (82), promoting leukemia stem cell survival and proliferation (77). Although exogenous interferon reduces *in vitro* self-renewal induced by RUNX1-ETO and RUNX1-ETO9a, interferon and interferon-stimulated genes are elevated by RUNX1-ETO in human and in murine models (83). Finally, the chromatin modifier TET2 is required for emergency myelopoiesis (84), and TET2 mutants show greater fitness in inflammatory environments partially due to increased resistance to TNF, which triggers IL6 overproduction and activation of an anti-apoptotic lncRNA (85, 86). Taken together, these studies link myeloid disorders with inflammation and indicate that AML may use a spectrum of different strategies for managing inflammatory signals.

## Interferon Treatment in AML

The interferon pathway is central to the inflammatory gene expression network, and it is heavily deregulated in cohesin-deficient macrophages (34). The deregulation of upstream interferon regulators like STAT1 and IRF7 disrupts basal interferon secretion, which maintains anti-viral transcriptional responsiveness by auto- and paracrine feed-forward signaling (87, 88). In the absence of cohesin, STAT and IRF-dependent enhancers fail to be induced, and consequently most interferon-induced genes are deregulated. Importantly, both enhancer activation and constitutive interferon gene expression can be partially rescued with exogenous interferon (34). These findings provide grounds to speculate that cohesin-mutated AMLs could be particularly vulnerable to interferon treatment. Mechanistically, supplying exogenous interferon could partially rescue expression of upstream transcription factors and regulators of the pathway, enabling normal enhancer activation, and transcription of downstream effectors. This would in turn increase the inflammatory responsiveness of cohesin-mutant AML cells, potentially restoring the balance between self-renewal and differentiation and restricting their selective advantage.

There is a long history of using type I interferons to treat hematological malignancies, with varying degrees of success. While early studies were hampered by treatment limiting side-effects, more recent recombinant and pegylated preparations are tolerated much better and can be used with feasible dosing regimens. There are currently established roles for interferon in the myeloproliferative disorders (89, 90), hypereosinophilic syndromes (91), and chronic myeloid leukemia (CML) (92). Intriguingly in CML, interferon treatment appears to preferentially target the leukemic stem cell population, and can induce cytogenetic remissions, some of which are durable upon treatment withdrawal, suggesting that it can cure some patients (92). Similar effects are observed in the JAK2 myeloproliferative disorders, with reduction or clearance of the mutant clones in up to 50% of patients (93).

In acute leukemia, interferon treatment impairs proliferation of AML cell lines *in vitro*, and has anti-leukemic effects in patient-derived xenograft models (PDX) in a dose dependent manner (94). This has been explained by cell intrinsic effects of interferon on leukemic blasts (reduced proliferation, increased apoptosis, and reduced secretion of growth-promoting cytokines), increased immunogenicity of interferon-treated leukemic blasts, as well as immunomodulatory effects on the residual normal hematopoietic cells, and increased clearance by the host immune system. However, despite the encouraging pre-clinical data, the clinical outcomes in interferon trials in AML have been disappointing, with durable responses seen in only small percentage of patients. While patients with secondary AML arising from a myeloproliferative disorder seem most susceptible, this is not exclusively the case. However, much of the clinical experience pre-dates the availability of current sequencing technologies and so stratification of AML by mutation may reveal genetic susceptibilities to interferon treatment.

## Conclusions

Many recently identified AML mutations are in genes encoding regulators of transcription and chromatin state. Understanding how these mutations are beneficial to cancer cell fitness is a major challenge (10). Regulators of transcription and chromatin state usually regulate the expression of hundreds or thousands of genes, which complicates the task of pinpointing the target genes that are responsible for the increased fitness of mutated cells.

Mutations in subunits of the cohesin complex result in clear alterations in the hematopoietic stem cell compartment and in HSPC function (29–33, 45). However, the specificity of cohesin control on HSPC gene expression has been difficult to accommodate with current models of cohesin function.

The transcriptional control of inducible gene expression provides a possible explanation for the high frequency of cohesin mutations in myeloid malignancies. Inflammatory signaling promotes the differentiation of HSPCs toward a myeloid fate (59), and cohesin-deficient cells show increased resistance to these differentiation-inducing stimuli (34, 45). In bone marrow microenvironments with alterations in cytokine levels such as those found in aging (70), myelodysplastic syndrome (MDS) (95) or leukemia (75, 76), mutations that confer reduced responsiveness to differentiation-inducing signals are likely to be positively selected and clonally expanded (45). This is consistent with observations that cohesin mutations appear early in the history of AML (42), and that cohesin mutations by themselves alter the composition of the HSPC compartment but are insufficient to trigger AML (31).

AML has an inherently poor prognosis, and even with intensive chemotherapy or hematopoietic stem cell transplantation the risk of relapse remains high. AML is a highly heterogeneous disease by morphological, clinical, and genetic criteria, underlining the need for targeted approaches. For a subset of recurrent mutations, such as FLT3, specific inhibitors are in clinical use (2). For others, like cohesin mutations, greater understanding of the molecular circuitries involved in the increased fitness of mutated cells is necessary to find vulnerabilities and new therapeutic approaches.

## Author Contributions

All authors listed have made a substantial, direct and intellectual contribution to the work, and approved it for publication.

### Conflict of Interest Statement

The authors declare that the research was conducted in the absence of any commercial or financial relationships that could be construed as a potential conflict of interest.
